# Prevalence and Risk Factors of Bacterial Pathogens Causing Camel Calf Diarrhea in Shabeley and Kebribayah Districts, Fafan Zone, Eastern Ethiopia

**DOI:** 10.1155/vmi/5519712

**Published:** 2025-12-29

**Authors:** Dek Kahin Yosef, Abdullahi Adan Ahad, Hassan Abdi Arog

**Affiliations:** ^1^ Department of Veterinary Microbiology and Public Health, College of Veterinary Medicine, Jigjiga University, Jigjiga, Somali Regional State, Ethiopia, jju.edu.et; ^2^ Department of Clinical Studies, College of Veterinary Medicine, Jigjiga University, Jigjiga, Somali Regional State, Ethiopia, jju.edu.et

**Keywords:** bacteriological, camel calf, diarrhea, Ethiopia, identification, isolation, risk factor

## Abstract

Infectious diarrhea is one of the most serious health threats to camel calves, causing death and substantial loss. This study aimed to determine the prevalence of bacterial pathogens causing diarrhea in camel calves and identify the associated risk factors in the Shabeley and Kebribayah Districts of the Fafan zone, Eastern Ethiopia. A cross‐sectional study was conducted from March 2022 to January 2023 to isolate and identify bacteriological infections of camel calf diarrhea and their associated risk factors in selected districts of the Fafen zone of the Somali region, Eastern Ethiopia. In total, 384 fecal swabs were collected from diarrheal, convalescent, and healthy camel calves. Using pure culture and bacteriological isolation, the overall prevalence of a bacterial infection was 66 (17.2%; 95% Cl: 0.13–0.21). Similarly, the distribution of the identified bacterial species was as follows: *Escherichia coli* (58%), *Salmonella* spp. (30%), and *Enterococcus* spp. (12%). The prevalence of camel calf infections in Kebribayah (9.0%) was higher than that in Shabeley (8.3%). Similarly, calves aged 7–12 months and diarrheal calves showed higher infection rates (7.6% and 8.6%, respectively). However, younger calves aged 0–3 months and apparently healthy calves had the lowest prevalence (4.7% and 3.4%, respectively). In the univariate logistic regression analysis, calves aged 7–12 months and calves with diarrhea showed statistically significant differences (*p* < 0.05). Furthermore, the incidence of diarrhea in camel calves was statistically significant (*p* > 0.05) in the multivariable logistic analysis. However, convalescent calves (OR = 1.79, 95% Cl: 0.38–1.64) were 1.79 times more likely to be infected with bacterial species than apparently healthy camel calves. This study indicated the presence of enteric bacteria in the study areas; therefore, further epidemiological investigations on other species of enteric bacteria and the implementation of public health education are warranted.

## 1. Introduction

Camels are among the most important livestock species in the arid and semi‐arid regions of the Horn of Africa, particularly in the pastoral and agro‐pastoral communities of eastern Ethiopia [1, 2]. They provide critical socioeconomic support by supplying milk, meat, transportation, and income [3]. In these regions, camels have become increasingly vital in the face of climate change and recurrent droughts because of their remarkable ability to survive harsh environments with minimal water and forage [4]. However, camel production is constrained by several health challenges, with neonatal and juvenile diseases posing significant threats to herd productivity and sustainability [5].

Diarrhea is a major health problem affecting young camel calves, leading to high morbidity and mortality rates during the first year of life, which threatens the future productivity of the herd [6]. This syndrome adversely affects calf growth rates, increases economic losses due to treatment costs and reduced production, and imposes additional labor burdens on pastoral households [7]. The etiology of diarrhea in camel calves is multifactorial, involving infectious agents such as bacteria, viruses, and parasites, as well as environmental and management factors [8, 9]. Among bacterial pathogens, *Escherichia coli*, *Salmonella* spp., and *Enterococcus* spp. are frequently implicated as primary causative agents of diarrhea in camel calves [10–12]. These bacterial infections are often exacerbated by poor hygiene, inadequate colostrum intake, malnutrition, and stress [10–12].

Despite the importance of camel rearing in the Somali Regional State of Ethiopia, epidemiological data on bacterial diarrhea in camel calves are scarce. Most existing studies focus on adult camels or other livestock species, leaving a significant knowledge gap regarding the prevalence, risk factors, and bacterial etiology of diarrhea in young camel calves in this region [13–15]. In particular, limited research has been conducted in the Shabeley and Kebribayah Districts, areas characterized by extensive camel production systems but lacking targeted investigations into enteric bacterial infections in calves.

Addressing this gap is critical for designing effective prevention and control strategies to reduce calf mortality, improve animal welfare, and enhance productivity in pastoral communities in the future. Therefore, this study aimed to determine the prevalence of bacterial pathogens causing diarrhea in camel calves and identify the associated risk factors in the Shabeley and Kebribayah Districts of the Fafan zone in Eastern Ethiopia. The findings will provide essential baseline information to support veterinarians, researchers, and policymakers in developing evidence‐based interventions tailored to the local epidemiological context of the disease.

## 2. Materials and Methods

### 2.1. Description of the Study Areas

**Figure 1:**
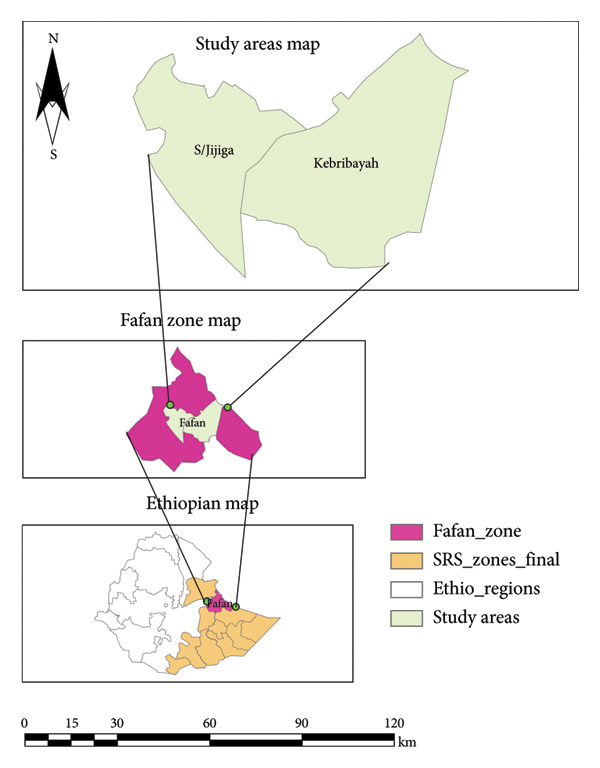
Map of the study areas.

The study was conducted in two purposely selected areas (Shabeley and Kebribayah) of the Fafan zone of Jigjiga, as shown in Figure 1, which are 7 and 50 km away from Jigjiga town, respectively. The zone is characterized by diverse topography, with approximately 52.6% of the area being plains, 31% hills, and 7% mountains. The elevation of the region ranges from 500 to 1650 m above sea level, with average minimum and maximum temperatures varying between 16°C–20°C and 28°C–38°C, respectively. Rainfall in the region is irregular, with an average annual rainfall of 600–700 mm. The zone is home to livestock production systems, mobile pastoral herds that roam across vast areas in search of pasture and water, and agro‐pastoral herds maintained by village residents who have limited mobility unless affected by drought or other circumstances [16].

### 2.2. Study Animals

The calves were local breeds maintained under a traditional extensive management system in two selected districts: Shabeley and Kebribayah. Based on suckling reflex and history, the subjects were classified as seemingly healthy, convalescent, or clinically infected, according to [17], and age was categorized as 0–3, 4–6, and 7–12 months, which were studied during the study. Finally, special emphasis was placed on the mucous membranes, the skin turgor test to check hydration status, and the inspection of the perianal area for signs of diarrhea.

### 2.3. Study Design

A cross‐sectional study was conducted from March 2022 to January 2023 to isolate and identify bacteriological infections of camel calf diarrhea and its associated risk factors from selected districts of the Fafen zone of the Somali region, Eastern Ethiopia. The risk factors considered in this study included age, sex, management practices, hygiene conditions, and environmental factors that could contribute to the occurrence of diarrhea in camel calves.

### 2.4. Sampling Techniques and Sample Size Determination

The two districts were purposively selected based on their dromedary camel production potential, previous outbreaks of camel calf infection, population, accessibility to transportation, proximity to livestock markets, and availability of animal‐watering wells. Subsequently, simple random sampling was used to select the Kebeles after obtaining the list from the District Livestock Bureau. The sampling frame was then established, and six kebeles were selected using random sampling techniques. Finally, systemic random sampling was used to select camel calves from herds within the selected kebeles.

The desired sample size for the study was determined considering a 50% expected prevalence, as there was no previous investigation on the prevalence of bacterial infections of camel calf diarrhea in the study districts, a 95% confidence level CI, and a 5% marginal error. The sample size was calculated as described by [18]

(1) n=Zα/22∗P1−Pd2.

The calculated sample size was 384 participants.

### 2.5. Sample Collection Method

Before obtaining samples from camel calves, information such as age, sex, breed, and clinical manifestations was recorded. Fecal samples were directly collected from the rectum of the animals in sterile plastic bags and kept on ice until they reached the laboratory for analysis. For bacterial testing, tagged feces were placed in individual stool caps and maintained in an icebox. On the day of collection, samples were sent to the Jigjiga University Veterinary Diagnostic Laboratories and Veterinary Regional Laboratory for rapid incubation at +4 for 24 h.

### 2.6. Laboratory Examinations

Fecal samples were spread across multiple types of growth media, including nonselective, moderately selective, and highly selective media. These include blood agar, neutral agar, MacConkey agar, aesculetin bile azide (ABA) agar, and *Salmonella*–*Shigella* agar (SSA). The goal of this study was to isolate, cultivate, and differentiate between various strains of *Salmonella* and *Shigella*. A single colony was obtained from the different types of colonies from the positive cultures and subcultured onto blood agar plates. This procedure was used to obtain pure cultures for further analyses and identification. The colonies were identified and classified based on their morphology. To further examine the colonies, Gram staining was performed under a light microscope at 100X magnification. Additionally, Gram‐negative bacteria were subcultured on MacConkey agar plates for further examination. [19]. Colony morphology was used for the initial identification of bacteria.

### 2.7. Data Analysis

The data were entered into a Microsoft Excel 2021 spreadsheet and subsequently transferred to STATA 17 for statistical analyses. Descriptive statistics were used to summarize the results of this study. Percentages were used to express the abundance of each species relative to the total number of isolates. Univariate logistic regression analysis was performed to assess the association between putative risk factors and the occurrence of diarrhea. The odds ratio was used to determine the degree of risk associated with diarrhea, with a 95% confidence interval providing an indication of the same. Variables with *p* < 0.05 in the univariate logistic regression analysis were subjected to multivariate logistic regression analysis to control for potential confounding variables. The goodness‐of‐fit of the model was tested using backward elimination, where variables were removed sequentially, starting from the least contributing variable, until the deletion of a variable significantly reduced the explained variable on the dependent variable. Additionally, collinearity between variables was checked using the standard error, and model fitness was ensured using the Hosmer–Lemshow and Omnibus tests. Throughout the data presentation, the confidence level was set at 95%, and a *p* value less than 0.05 (i.e., statistical significance was set at *p* < 0.05) was considered statistically significant.

### 2.8. Ethical Considerations

This study was approved by the Ethics Committee of Jigjiga University College of Veterinary Medicine (approval number: JJU/CVM/032/2023), and permission letters were obtained from appropriate authorities. All animal owners provided written and oral consent for voluntary participation.

## 3. Result

### 3.1. Distribution of Isolated Enteric Bacterial Species in Camel Calves

**Figure 2:**
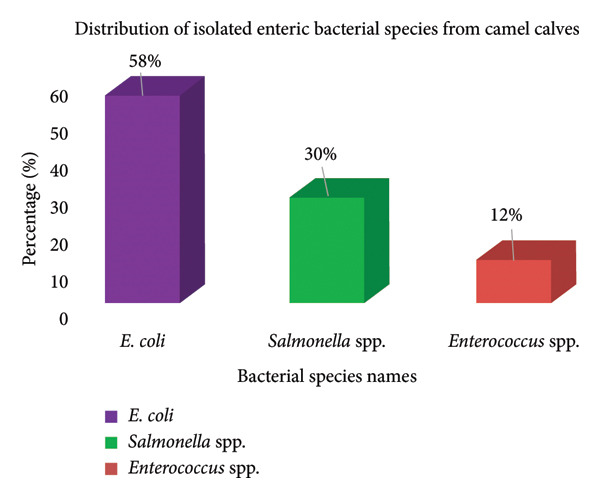
Distribution of isolated enteric bacterial species from camel calves in the Shabeley and Kebribayah Districts, Fafan zone, Eastern Ethiopia.

The distribution of enteric bacterial pathogens isolated from diarrheal, convalescent, and apparently healthy camel calves revealed significant findings. *E. coli* was the predominant bacterium, accounting for 58% of the total isolates among the 66 positive cases. *Salmonella* spp. and *Enterococcus* spp. accounted for 30% and 12% of the isolates, respectively. The prevalence of *E. coli* underscores its role as a primary etiological agent of camel calf diarrhea within the study area, suggesting potential fecal‐oral transmission and suboptimal hygiene practices. The comparatively lower isolation rates of *Salmonella* and *Enterococcus* spp. may indicate sporadic outbreaks or variations in pathogenicity and environmental survival (Figure 2).

### 3.2. Demographic and Clinical Factors Influencing Bacterial Infection

An analysis of bacteriological infections in camel calves stratified by location, sex, age, and health status yielded noteworthy results. The Kebribayah District exhibited a marginally higher infection rate (9.0%) than Shabeley (8.3%), which is potentially attributable to environmental or herd‐management disparities. Male calves demonstrated a slightly higher infection rate (10.0%) than female calves (7.3%), possibly reflecting behavioral exposure differences. In contrast, calves aged 0–3 months exhibited the highest prevalence (7.6%), followed by the 4–6‐month (5.0%) and 7–12‐month (4.7%) groups, indicating heightened susceptibility in younger calves due to their immature immune systems. Health status emerged as a crucial differentiating factor, with apparently healthy calves unexpectedly showing an 8.6% prevalence, potentially indicative of subclinical infections or early‐stage colonization. Diarrheic calves exhibited 5.2% positivity, whereas convalescent calves had the lowest positivity at 3.4%, suggesting ongoing exposure and potential bacterial shedding even in nonclinical animals (Table 1).

**Table 1:** Prevalence of bacterial infection in relation with potential demographic factors of camel calf diarrhea.

Variables	Category	Sample tested (%)	Sample positive (%)
Site	Kebribayah	213 (55.5)	34 (9.0)
	Shabeley	171 (44.5)	32 (8.3)

Sex	Female	145 (37.8)	28 (7.3)
	Male	239 (62.2)	38 (10.0)

Age (months)	0–3	115 (30)	29 (7.6)
	4–6	144 (37.5)	19 (5.0)
	7–12	125 (32.6)	18 (4.7)

Health status	Apparently, health	164 (42.7)	33 (8.6)
	Diarrheic	76 (19.8)	20 (5.2)
	Convalescent	144 (37.5)	13 (3.4)

### 3.3. Univariable Logistic Regression Analysis of Risk Factors

Univariate logistic regression analysis evaluating the associations between demographic and clinical factors and the presence of bacterial infection revealed significant findings. Although site and sex did not show statistically significant associations, age and health status were notable factors. Calves aged 0–3 months demonstrated significantly higher odds of bacterial infection than the 7–12‐month reference group (OR = 2.00, 95% CI: 1.04–3.85, *p* = 0.037), supporting the hypothesis that immunological immaturity is a strong risk factor. Regarding health status, diarrheic calves were significantly less likely to be bacterially infected than apparently healthy calves (OR = 0.47, 95% CI: 0.25–0.85, *p* = 0.014), suggesting a paradoxical relationship between clinical signs and active bacterial infection, possibly because of recent antimicrobial use or recovery phase dynamics (Table 2).

**Table 2:** Univariable logistic regression analysis of factors associated with bacteriological infection among camel calf diarrhea.

Variables	Category	No tested (%)	No positive (%)	OR (95% Cl)	*p* value
Site	Kebribayah	213 (55.5)	34 (9.0)	Ref.	
	Shabeley	171 (44.5)	32 (8.3)	1.21 (0.71–2.06)	0.480

Sex	Female	145 (37.8)	28 (7.3)	Ref.	
	Male	239 (62.2)	38 (10.0)	0.80 (0.46–1.35)	0.391

Age (months)	0–3	115 (30)	29 (7.6)	2.00 (1.04–3.85)	**0.037** ^**∗**^
	4–6	144 (37.5)	19 (5.0)	0.90 (0.45–1.81)	0.780
	7–12	115 (30)	29 (7.6)	Ref.	

Health status	Apparently, health	164 (42.7)	33 (8.6)	Ref.	
	Diarrheic	76 (19.8)	20 (5.2)	0.47 (0.25–0.85)	**0.014** ^**∗**^
	Convalescent	144 (37.5)	13 (3.4)	1.16 (0.64–2.02)	0.515

*Note:* Bold values indicate statistically significant results. An asterisk (∗) denotes statistical significance at *p* < 0.05.

### 3.4. Multivariable Logistic Regression Analysis of Risk Factors

The multivariable logistic regression model, adjusted for potential confounders, provided further insights. Although calves aged 0–3 months still exhibited higher odds of bacterial infection (OR = 1.8, 95% CI: 0.92–3.49), this association was no longer statistically significant (*p* = 0.088), indicating that the effect of age may be modulated by other variables in a multivariable context. Health status remained a significant predictor, with calves with diarrhea showing significantly lower odds of infection (OR = 0.49, 95% CI: 0.27–0.92, *p* = 0.026), reinforcing the univariable result. This counterintuitive finding may suggest prior exposure, recent antimicrobial treatment, or misclassification as nonbacterial diarrhea. The convalescent group showed no significant association (OR = 1.79, 95% CI: 0.38–3.64, *p* = 0.831). These multivariate results underscore the complexity of the epidemiology of bacterial diarrhea in camel calves and highlight the need for comprehensive disease management and prevention strategies (Table 3).

**Table 3:** Multivariable logistic regression analysis of factors associated with bacteriological infection among camel calf diarrhea.

Variables	Categories	No tested (%)	No positive (%)	OR (95% Cl)	*p* value
Age (months)	0–3	213 (55.5)	34 (9.0)	Ref.	
	4–6	171 (44.5)	32 (8.3)	0.81 (0.40–0.65)	0.575
	7–12	145 (37.8)	28 (7.3)	1.8 (0.92–3.49)	0.088

Health status	Apparently, health	164 (42.7)	33 (8.6)	Ref.	
	Diarrheic	76 (19.8)	20 (5.2)	0 0.49 (0.27–0.92)	**0.026** ^**∗**^
	Convalescent	144 (37.5)	13 (3.4)	1.79 (0.38–3.64)	0.831

*Note:* Bold values indicate statistically significant results. An asterisk (∗) denotes statistical significance at *p* < 0.05.

## 4. Discussion

This study examined 384 camel calves and found an overall prevalence of bacterial infection of 17.2% (66 cases). *E. coli* was the predominant pathogen, accounting for 58.0% of infections. This finding aligns with previous studies from Saudi Arabia, Ethiopia, and Tunisia, which reported *E. coli* prevalence ranging from 52.8% to 59% [20–22]. However, our results were higher than those reported in several other studies, which found prevalence rates between 5.7% and 28.2% [21, 23–26]. Conversely, our findings were lower than the 65.9% prevalence reported in Ethiopia [27]. The higher prevalence of *E. coli* infections in camel calves in this study compared to that in other studies may be attributed not only to geographical and herd management differences but also to potential variations in the virulence of *E. coli* strains present in the study area. Environmental factors, such as climate, sanitation, and water quality, as well as genetic differences in local camel populations, may also influence susceptibility and pathogen dynamics. Further molecular characterization of *E. coli* isolates, including serotyping and virulence gene profiling, would provide deeper insights into these hypotheses by identifying specific pathogenic strains and their epidemiological significance.

*Salmonella* spp. was the second most prevalent pathogen at 30.0%, which is higher than that reported previously, ranging from 13.2% to 20.1% [28, 29]. The increased incidence of *Salmonella* species may be due to overcrowding and transportation stress in animals, as well as environmental conditions that favor bacterial survival and transmission. The prevalence of *Enterococcus* spp. was 12.0%, which is consistent with some studies [21, 30] but higher than that of others [20].

Several risk factors for bacterial infections in camel calves with diarrhea have been identified. Age was a significant factor, with calves aged 0–3 months showing the highest prevalence. This likely reflects their immunological immaturity, including underdeveloped innate and adaptive immune responses, limited exposure to environmental antigens, and dependence on passive immunity from colostrum, which may be inadequate or delayed in some cases [31]. These physiological vulnerabilities increase susceptibility to bacterial colonization and infections. Health status also played a crucial role, with diarrheic calves being nearly twice as likely to be infected by bacterial pathogens. This finding aligns with those of studies conducted in Korea, the United States, and Ethiopia [21, 32, 33]. This association may be explained by compromised gut barrier function and altered microbiota during diarrheal episodes, facilitating pathogen proliferation. Conversely, the paradoxical finding of lower bacterial detection in diarrheic calves than in apparently healthy calves could indicate prior antimicrobial treatment, transient bacterial clearance, or the presence of nonbacterial causes of diarrhea, highlighting the complexity of clinical presentations.

Although site and sex did not show statistically significant associations, slight differences in prevalence suggested that herd management practices, hygiene, water quality, or nutritional differences might still influence infection rates. For example, the marginally higher infection rate in male calves (10.0%) compared to that in female calves (7.3%) could be linked to behavioral factors and hormonal influences on immune function, which merit further investigation. Further investigations with larger sample sizes are required to confirm this hypothesis.

The analysis revealed that the Kebribayah District exhibited a slightly higher infection rate (9.0%) than Shabeley (8.3%). This difference may be influenced by variations in environmental conditions, such as climate and sanitation, and differences in herd management practices between the two districts. For instance, Kebribayah might have factors such as higher humidity or closer proximity to water sources that favor bacterial proliferation, or less stringent biosecurity measures that increase exposure risk.

Regarding sex‐based differences, male calves showed a marginally higher infection rate (10.0%) than female calves (7.3%). This disparity could be attributed to behavioral and physiological factors; male calves may engage in more exploratory or aggressive behaviors, increasing their contact with contaminated environments or infected peers. Additionally, hormonal differences may influence immune responses, potentially rendering males more susceptible to infections.

By stratifying infection rates according to these factors, this study provides a nuanced understanding of the epidemiology of bacteriological infections in camel calves, highlighting the importance of tailored management interventions that consider local environmental conditions, host physiology, and pathogen characteristics.

Univariable logistic regression analysis revealed that calves aged 7–12 months had twice the odds of bacterial infection compared to those aged 0–3 months (OR = 2.00; 95% CI: 1.04–3.85; *p* = 0.037). This finding contradicts the general trend of decreasing infection rates with age, possibly because of specific environmental or management factors in the study area. Diarrheic calves were significantly more likely to be infected compared to apparently healthy calves (OR = 0.47; 95% CI: 0.25–0.85; *p* = 0.014), consistent with a study conducted in Saudi Arabia [20].

A notable limitation of this study was the absence of serotyping and molecular characterization of *E. coli* and *Salmonella* isolates. Incorporating these analyses in future research will enable the precise identification of virulent strains, assessment of antimicrobial resistance (AMR), and elucidation of transmission dynamics, thereby strengthening epidemiological understanding and informing more effective control measures.

We have emphasized the limitation related to the scope of risk factors included in this study, which primarily focused on key demographic and clinical variables, such as age, sex, health status, and location. We acknowledge that the narrow focus on demographic and clinical variables constrains the depth of epidemiological insights. Future studies should include detailed management practices, environmental assessments, nutritional status, and antimicrobial use to provide a more comprehensive epidemiological framework. Such multidimensional approaches are essential for unraveling the complex interactions driving bacterial diarrhea in camel calves and developing targeted, evidence‐based prevention and control strategies.

Future studies should be designed to build on the current findings and provide a deeper and more comprehensive understanding of camel calf diarrhea. Molecular characterization of isolated bacterial strains through whole‐genome sequencing to identify virulence factors and AMR genes, as well as phylogenetic analysis to understand the genetic relationships between the isolates. Expanded pathogen screening includes testing for viral and parasitic causes of diarrhea in addition to bacteria and investigation of co‐infections and their impact on disease severity. Longitudinal studies should follow calves over time to assess long‐term health impacts and recurrence of infections and evaluate the effectiveness of different management practices in reducing the incidence of diarrhea. AMR profiling was performed by antibiotic susceptibility testing on isolated pathogens and monitoring trends in resistance patterns over time. Immunological studies are needed to assess the immune responses and development in camel calves and to investigate the potential for vaccine development against major pathogens. Economic impact analysis was used to quantify production losses and treatment costs associated with calf diarrhea and to evaluate the cost‐effectiveness of different prevention and control strategies. A One Health approach to investigate the zoonotic potential of isolated pathogens and assess the risk factors for transmission between camels, other livestock, and humans. Microbiome studies were conducted to characterize the gut microbiome composition in healthy and diarrheic calves and explore the potential for probiotic interventions. Environmental sampling was used to test water sources, soil, and feed for the presence of pathogens and identify environmental reservoirs and transmission routes. The geographical scope should be expanded to conduct similar studies in other camel‐rearing regions for comparison and to investigate regional variations in pathogen prevalence and risk factors. These additional research directions will provide a more comprehensive understanding of camel calf diarrhea and inform evidence‐based prevention and control strategies for this condition.

## 5. Conclusion and Recommendation

In this study, the overall prevalence of bacterial infections in camel calves was 17.2%, with *E. coli* (58%), *Salmonella* spp. (30%), and *Enterococcus* spp. (12%) as the predominant pathogens. The significant associations found between bacterial infections and both diarrheic conditions and the 0–3‐month age group underscore the vulnerability of young calves and the clinical relevance of these pathogens. Given the zoonotic potential of these bacteria and the growing concerns regarding AMR, this study emphasizes the critical need for a One Health approach that integrates animal, human, and environmental health. Such an approach would facilitate the development of comprehensive disease management strategies that not only improve camel calf health and productivity but also mitigate public health risks. Therefore, targeted community education focusing on hygiene, early disease recognition, and improved calf‐rearing practices is recommended for camel herders and local veterinarians. Additionally, implementing vaccination programs against the most prevalent pathogens and establishing antibiotic stewardship protocols tailored to the local context are essential to curb AMR. Ongoing surveillance, incorporating molecular characterization and AMR profiling, should be prioritized to inform evidence‐based interventions. Expanding future research to include environmental and management‐related risk factors, as well as exploring zoonotic transmission dynamics, will further strengthen control efforts and promote sustainable livestock and public health outcomes in the region.

Future research should include comprehensive serotyping and molecular characterization of *E. coli* and *Salmonella* isolates to enable precise identification of virulent strains, assessment of AMR, and elucidation of transmission dynamics of these pathogens. This will strengthen the epidemiological understanding and inform more effective control measures. Based on the significant association between diarrheic conditions and bacterial infections in camel calves observed in this study, targeted community education programs focusing on hygiene management, early disease recognition, and improved calf‐rearing practices are recommended for camel herders and local veterinarians. Furthermore, the development and implementation of vaccination programs against the most prevalent pathogens identified could provide sustainable reduction in disease incidence. Additionally, establishing antibiotic stewardship protocols tailored to the local context will help mitigate AMR. These evidence‐based interventions, combined with ongoing surveillance, will enhance disease control and improve the health outcomes of camel calves. The study acknowledges the limitation of the scope of risk factors assessed, which were primarily demographic and clinical variables, such as age, sex, health status, and location; future studies should expand on these to include environmental and management‐related factors. Finally, further studies on the major infectious causes of camel calf diseases and public education are required.

## Conflicts of Interest

The authors declare no conflicts of interest.

## Author Contributions

Dek Kahin Yosef: data collection, laboratory work, conceived the original, and wrote the first manuscript. Abdullahi Adan Ahad: data analysis and final manuscript preparation. Hassan Abdi Arog: conceptualization, design, and sample collection.

## Funding

No funding was received for this manuscript.

## Data Availability

All data generated or analyzed in the present study can be obtained from the corresponding author upon request.
